# Multiannual Assessment of Quality of *Plantago major* L. Seed Progeny from Kyshtym Radiation Accident Area: Weather-Dependent Effects

**DOI:** 10.3390/plants12132528

**Published:** 2023-07-02

**Authors:** Nadezhda S. Shimalina, Elena V. Antonova, Vera N. Pozolotina

**Affiliations:** Institute of Plant and Animal Ecology, Ural Branch of Russian Academy of Sciences, 8 Marta Str. 202, Ekaterinburg 620144, Russia; selena@ipae.uran.ru (E.V.A.); pozolotina@ipae.uran.ru (V.N.P.)

**Keywords:** greater plantain, East Ural Radioactive Trace, radioactive contamination, seed progeny, viability, radioresistance, low dose, weather conditions, precipitation, combined effects

## Abstract

The effects of low-dose radiation that are observed in plant populations in radioactively contaminated areas are variable. One of the reasons is the influence of fluctuating weather conditions and the interaction of radiation with weather factors. This article summarizes results of 12-year research on the viability and radioresistance of greater plantain (*Plantago major* L.) seed progeny growing in the East Ural Radioactive Trace (EURT) zone and in control (nonradioactive) areas, with consideration of weather conditions’ variability. The EURT was formed by the Kyshtym accident, which occurred in 1957 at the Mayak Production Association. Absorbed dose rates of *P. major* parental plants in the pollution gradient were 14.5–165.9 μGy h^−1^, which correspond to a low-dose range. Seed progeny quality was evaluated as seed weight, the survival rate, and root length of 21-day seedlings. Interannual variability in the studied parameters was high, and their ranges overlapped between EURT groups of seeds and control groups in most cases. The number of significant correlations between the parameters of seed quality and weather conditions was higher in EURT groups than in control populations. In the control groups of seeds, 88.9% of correlations were negative, whereas in the EURT groups, 78.5% were positive.

## 1. Introduction

Severe accidents at nuclear industry facilities such as Kyshtym (1957), Chernobyl (1986), and Fukushima (2011) have resulted in radioactive contamination of vast territories [1,2]. These areas have become unique testing sites for studying radiobiological effects in the organisms and communities that have been exposed to radiation stress for generations [2,3]. The increased frequency of morphological anomalies and variations in viability, radiosensitivity, and biochemical parameters have been shown in natural plant populations under conditions of radioactive contamination [4,5,6]. An elevated level of mutagenesis that has persisted for many years has been observed in populations of many plant species in both the Kyshtym and Chernobyl accident areas [7,8,9,10]. In most of the studied cases, the dose–effect relationship has been nonlinear, and the yield of mutations per dose unit has been higher at low-dose and low-power irradiation [8]. The difference between the effects of low and high doses of ionizing radiation as well as the genetic basis of these phenomena and the dynamics of their persistence across generations remain insufficiently understood [11,12,13]. Evidence indicates the enhanced genetic and phenotypic plasticity of organisms under conditions of chronic exposure, i.e., the ability to respond in more diverse ways to habitual environmental exposures, which broadens the norm of the reaction in local populations [6].

Comprehensive radiobiological studies have revealed a variety of effects at all levels of biological organization, but there is still no consensus on the consequences of the chronic influence of low-dose irradiation on a biota [14,15,16,17]. One of the reasons for uncertainty in the manifestation of radiobiological effects may be the fact that natural populations simultaneously experience the effects of a combination of abiotic and biotic factors [18,19]. The interaction of low-dose irradiation with factors of a nonradioactive nature, in particular weather conditions, can modify the viability of seed progeny and phenotypic traits of organisms living under the conditions of radioactive contamination [3,6]. For instance, the effect of the weather-climatic factors on Scots pine from the Chernobyl zone has manifested itself as high interannual variability in seed viability [20]. Data on the influence of weather conditions on multiannual dynamics of seed progeny quality in some herbaceous plant species from the East Ural Radioactive Trace (EURT) indicate that the manifestation of the combined effects of these factors is species-specific [6,18,21,22]. It is necessary to expand the range of species of different families investigated in this way. Of particular interest are radiosensitive organisms, such as greater plantain (*Plantago major* L.), which is a widespread medicinal plant [23]. Our previous studies have revealed upregulation of an oxidative-stress marker called malondialdehyde and of the activity of antioxidant enzymes in seedlings of this species from the EURT zone [5]. In a random group from the most contaminated local population, the effects have persisted in subsequent generations of this species after the removal of the radiation load [12]. Microsatellite analysis has uncovered a decrease in genetic diversity of *P. major* from the same area as compared with control (background radioactivity) populations [24]. A database on the viability and radiosensitivity of *P. major* seed progeny, which has been compiled over 12 years of monitoring studies, is analyzed in this article, taking into account weather conditions.

The purpose of this study was to evaluate the combined effects of different weather-climatic factors and low doses of radiation on the interannual variability in seed weight and viability and on the radiosensitivity of seed progeny of *P. major*. On the basis of our own research and data from the literature, we hypothesized that (1) greater plantain seed quality does not depend on the absorbed dose rate of parental plants in a low-dose range; (2) the viability and radiosensitivity of seed progeny of this species from the EURT zone are more affected by weather factors as compared with those of background (nonradioactive zone) groups of randomly collected seeds.

## 2. Results

### 2.1. Interannual Variability in 1000-Seed Weight

**Figure 1:**
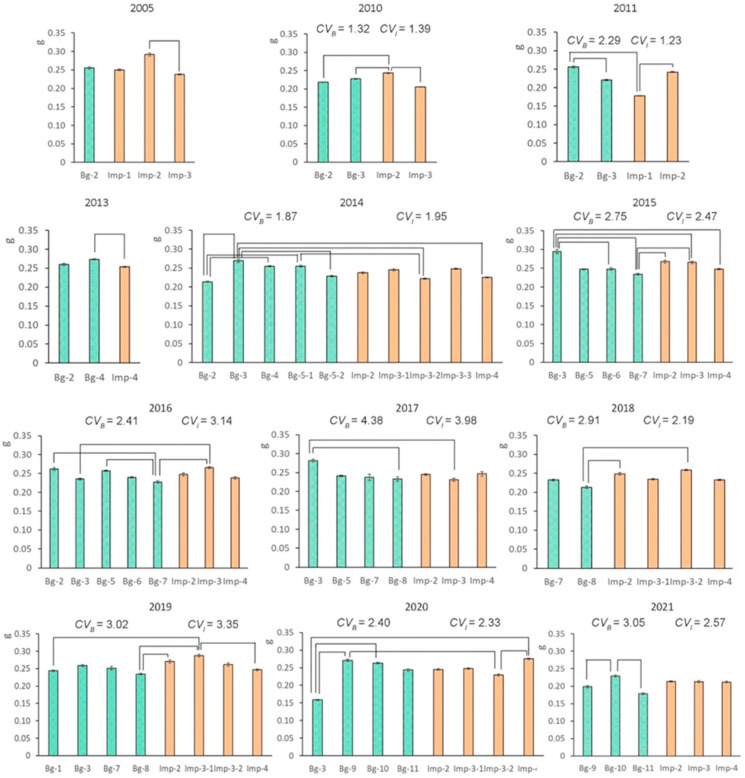
Variability in 1000-seed weight in *P. major* from impact groups of seeds and from background groups in different years (mean ± SE). Square brackets indicate significant differences in pairwise comparisons (Dunn’s test, *p* < 0.001 to 0.045). *CV_B_* and *CV_I_* are coefficients of variance of this parameter in the background and impact zones, respectively, not shown for years when there were fewer than two groups in any zone.

The average values of plantain 1000-seed weight over 12 years (Figure 1) in background groups of randomly collected seeds and in impact (radioactive zone) groups were 0.241 g (range: 0.159–0.294 g) and 0.243 g (0.178–0.291 g), respectively. Factor “site” had a significant effect on seed weight (*H*_(2–9; N = 15–50)_ = 10.7–45.8; *p* < 0.001 to 0.049) in all study years. This parameter showed low variability (coefficient of variance [*CV*] = 1.32–4.38). Seed weight in the total (pooled) EURT group was significantly higher than that in the pooled background group only in 2018 and 2019 (*U* test, *p* < 0.001) and was lower in 2013 (*p* = 0.014). No correlation was found between the seed weight and dose rates of parental plants (*R* = 0.033; *p* = 0.781).

Factor “year” had a significant effect on seed weight at most of the analyzed impact and background sites (*H*_(1–10; N = 10–75)_ = 6.8–54.9; *p* < 0.001 to 0.048), except for groups Bg-6 and Bg-7 (*H*_(1–4; N = 10–25)_ = 2.8–7.5; *p* = 0.094–0.112). Lightweight seeds formed in the EURT zone in 2011 (0.210 g) and in the background zone in 2021 (0.202 g). The greatest seed weights were observed in the impact and background zones in 2019 (0.266 g) and 2013 (0.267 g), respectively. The interannual variability in seed weight may apparently be related to differences in weather conditions during their formation.

### 2.2. Interannual Variations in Viability of Seed Progeny

**Figure 2:**
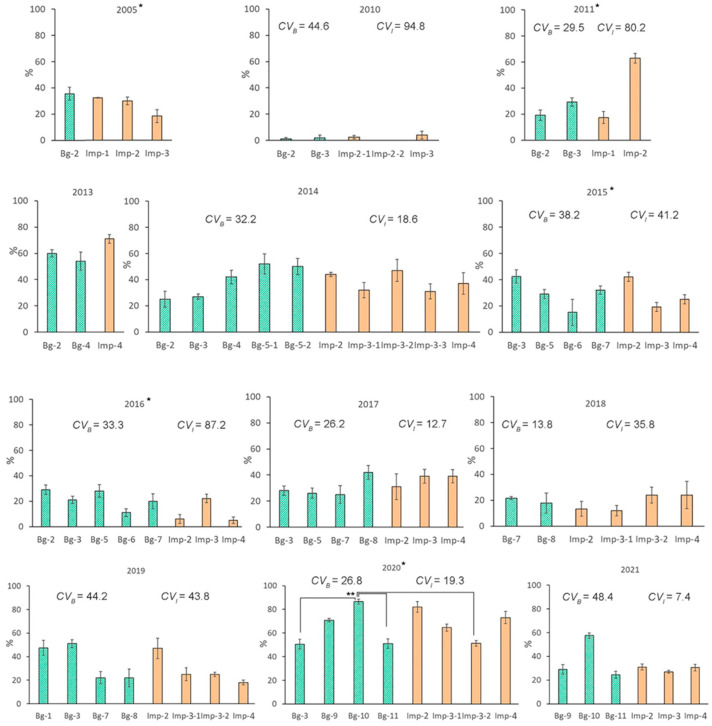
Variability in the survival rate of *P. major* seedlings from impact groups of seeds and from background groups in different years (mean ± SE). * A significant effect of factor “site” (*H*_(3–7; N = 12–32)_ = 8.0–20.1; *p* = 0.005–0.047). ** Dunn’s test, *p* = 0.022–0.026. *CV_B_* and *CV_I_* are coefficients of variance of this parameter in the background and impact zones, respectively, not shown for years when there were fewer than two groups in any zone.

The average survival rate of *P. major* seedlings over 12 years was 32.9% in the background groups and 28.8% in the impact groups. Seed progeny collected on the same plots in different years were found to have high interannual variability in survival rates. For instance, ranges of the variability of this parameter were similar between different zones (background: 1–86.5%, impact: 0–82%; Figure 2), i.e., the variability did not exceed the norm of the response.

The lowest values in both zones were observed in 2010, and the highest in 2020. In 2010 in the Urals, there was a severe drought, which was associated with hot weather. The Selyaninov hydrothermal coefficient (*HTC*) for the summer months at the weather station closest to the EURT was 0.3 and was the lowest among the 12 years of observations, whereas in other years, it varied from 1.0 to 1.9 (in 2020: *HTC*_6–8_ was 1.55) (Table A1). Coefficients of variance (*CV*) changed from 7.4% to 94.8% in the EURT area and from 13.8% to 48.4% in background groups of seeds. In 2010, 2011, 2016, and 2018, there was a twofold higher *CV* in the impact zone compared with the background. This finding indicated higher variability in seedling survival in the irradiated groups.

There was no significant effect of the “site” factor on seedlings’ survival rate (*H*_(2–9; N = 12–48)_ = 3.4–16.8; *p* = 0.052–0.633) within seven individual years of the study. Within the other five years (2005, 2011, 2016, 2019, and 2020), the “site” factor had a significant effect (*H*_(3–7; N = 12–32)_ = 8.1–20.1; *p* = 0.005–0.043). Differences between groups of seeds were found only in 2020 in the pairs Bg-10–Bg-11, Bg-10–Bg-3, and Bg-10–Imp-3 (Dunn’s test, *p* = 0.022–0.026).

The factor “year” had a significant effect on the seedling survival rate at all sites of the EURT (*H*_(1–10; N = 7–54)_ = 4.8–39.3; *p* < 0.001 to 0.028). Similar effects were noted at background sites Bg-2, Bg-3, Bg-9, Bg-10, and Bg-11 (*H*_(1–7; N = 8–57)_ = 5.4–33.0; *p* < 0.001 to 0.020). Analysis of groups pooled by zone showed that the seedling survival rate in the EURT zone was lower than that in the background area in 2016 (*U* test, *p* = 0.013) but higher in 2013 (*U* test, *p* = 0.025). In the other years, there were no significant differences between the pooled impact groups and pooled background groups. No significant correlation was observed between the seedling survival rate and parental-plant dose rates (*R* = −0.01; *p* = 0.921). There was no significant correlation between seed weight and seedling survival rate in either the impact (*R* = 0.18; *p* = 0.281) or background (*R* = 0.27; *p* = 0.098) zone.

**Figure 3:**
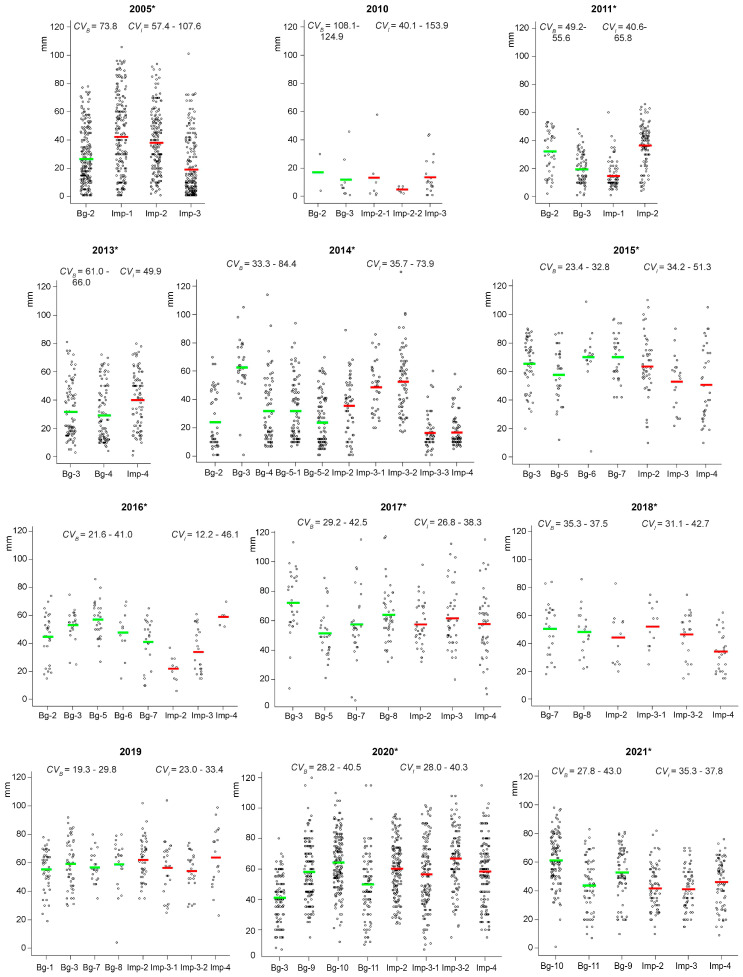
Variability in root length of *P. major* seedlings in the impact and background areas in different years (the dots indicate the root length of each seedling [mm], while the red and green marks represent the average values in the impact and background zone, respectively). * Years in which a significant effect of the factor “site” was found (*H*_(2–9; N = 45–1141)_ = 15.6–198.6; *p* < 0.005 to 0.008). Results of pairwise comparisons are given in Table A2, Table A3, Table A4, Table A5, Table A6, Table A7, Table A8, Table A9, Table A10 and Table A11. *CV_B_* and *CV_I_* are the ranges of variance coefficients of root length in the background and impact zones, respectively.

Similar data were obtained for the “root length” parameter (Figure 3). The lowest mean values of this trait were observed in 2010 (severe drought) in the EURT groups (5 mm) and in background groups (12 mm). The greatest average root lengths were recorded in 2017 in the background groups (72 mm) and in 2020 in the EURT zone (67 mm). The factor “year” had an effect on seedling root length in all plantain coenopopulations of the EURT area (*H*_(8–10; N = 543–767)_ = 213.1–301.9; *p* < 0.001) and in half of the background plots (Bg-2, Bg-3, Bg-6, Bg-7, and Bg-8; *H*_(1–7; N = 27–442)_ = 9.0–234.0; *p* <0.001 to 0.011). The absence of differences in root length between years was registered in those background coenopopulations where the time series of observations was short (2 to 3 years).

The variability in root length in some years was high. A significant effect of the “site” factor on root length was detected in most years, except for 2010 and 2019, and ranges of variability of this parameter overlapped between the background and impact groups (see Figure 3). Root length of the pooled EURT group was less than that of the pooled background group in 2015, 2016, 2018, and 2021 (*U* test, *p* < 0.001 to 0.048) and higher in 2005, 2013, and 2020 (*U* test, *p* < 0.001 to 0.026). Coefficients of variance in impact groups exceeded those in background groups in half of the years of observation. Root length also showed no significant correlations with the dose rates of parental plants (*R* = −0.02; *p* = 0.867). A significant correlation between seed weight and root length was observed in the EURT zone (*R* = 0.37; *p* = 0.025) but was not found in the background zone (*R* = 0.28; *p* = 0.081).

Thus, the comparison of the viability of *P. major* seed progeny between impact and background populations during the 12 years revealed a variety of effects: parameters were higher, lower, or not different. The seedling survival rate and root length were not affected by the dose loads of parental plants. Ranges of the variability of all parameters of seed progeny overlapped between the different zones in different years, and significant differences were found only within some pairs of impact and background groups. The range of variance coefficients for the survival rate of seedlings from the EURT zone exceeded that of background plots. Interannual variability in the seedling survival rate, seed weight, and root length was high.

### 2.3. Variability in Radioresistance of Seed Progeny from Different Zones

**Figure 4:**
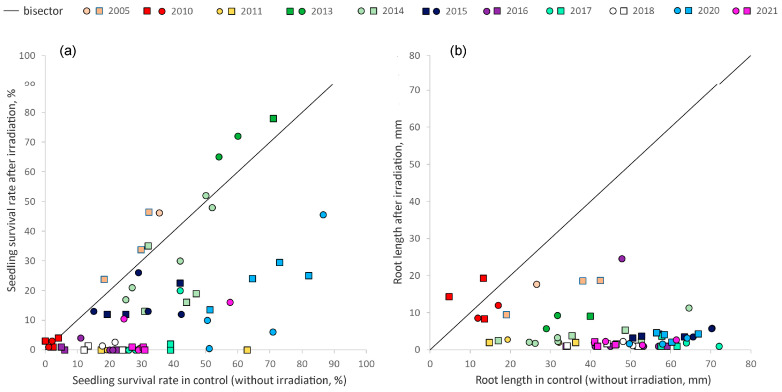
Average seedling survival rates (**a**) and root lengths (**b**) before and after irradiation at a dose of 100 Gy in different years. The line is the bisector. The squares denote EURT groups of seeds, and the circles background groups.

The survival rate of seedlings after pre-sowing irradiation of seeds by us at a dose of 100 Gy either diminished (in most groups under study) or did not differ from an identical unirradiated control (Table 1). Only in 2005 did the Imp-1 group show high radioresistance: the survival rate after the irradiation increased by 44% (*p* = 0.027). Seed progeny with a very low survival rate formed in 2010 under the conditions of an abnormal drought, and this parameter did not change after the additional irradiation. In 2005, 2010, 2013, 2014, and 2016, the survival rate after the irradiation positively correlated with the unirradiated control in both background groups (*R* = 0.86, *p* < 0.001) and impact groups (*R* = 0.82, *p* < 0.001). During the years in which there was significant suppression of survival, such a relation was weaker (*R* = 0.57–0.65, *p* = 0.008–0.009). Thus, the higher the seedling survival without irradiation, the fewer plants died after the seed irradiation (Figure 4a). Similar effects were registered after irradiation at a dose of 200 Gy, with the exception of the 2005 and 2014 harvests. During those periods, the higher dose severely slowed the formation of true leaves.

Root length proved to be the parameter most sensitive to the irradiation. Inhibition of root growth after 100 Gy irradiation was observed in most groups under study (see Table 1, Figure 4b). An exception was the year 2010′s groups from different zones, in which the root length of seedlings was low before and after irradiation; no differences from the unirradiated control were observed (*p* = 0.19–0.86). At the irradiation dose of 200 Gy, inhibition of root growth was even more pronounced; the range of average values decreased to 1–17 mm in the background and impact groups with controls at 17–72 and 5–67 mm, respectively. Negative correlations were detected between the parameters of irradiated and unirradiated seedlings within the background groups (*R* = −0.40 to −0.60, *p* < 0.001 to 0.01) and impact groups (*R* = 0.84, *p* < 0.001). That is, root growth slowed down in different years in both zones after the additional irradiation even in cases of substantial root lengths without irradiation. No significant correlations were found between the dose rate and seedling survival or root length after irradiation at different doses (*R* = −0.086 to −0.076; *p* = 0.488–0.867). Consequently, in the seed progeny of P. major from the EURT zone, no stable signs of pre-adaptation (i.e., enhanced resistance to additional acute irradiation) were found in plants chronically exposed to low radiation doses. The only case of increased radioresistance of seeds was population Imp-1 in 2005. This exception only confirms the rule about the instability of biological effects under the action of low doses of radiation.

### 2.4. The Effect of Weather Factors on Seed Weight

No dependence of greater plantain seed mass on weather parameters was revealed in the background zone. Seed mass that formed in the EURT zone positively correlated with *HTC* and with the sum of precipitation for August in the year of seed formation as well as with the sum of precipitation for October of the preceding year (Table 2).

### 2.5. The Influence of Weather Factors on the Viability of Seed Progeny

Table 3 lists the weather factors that had a significant influence on the viability of *P. major* seed progeny formed in the EURT zone and on background plots. A positive correlation between the sum of precipitation for August and the seedling survival rate was detected for groups from the EURT zone, as was a negative correlation between the sum of effective temperatures for April and the biological response.

Root length in the background groups negatively correlated with precipitation sums in September, November, and December of the preceding season and in March and June of the current season. Root length in the background groups also negatively correlated with the *HTC* in June. The *HTC* for April positively correlated with root length. Root lengths in seedlings from the EURT zone (in contrast to background groups) positively correlated with the sum of precipitation at effective temperatures and with the *HTC* in July and August. There were negative dependences of root length on the sum of effective temperatures in September and on the sum of precipitation in December of the preceding year.

Only two common weather-climatic factors (the sum of precipitation in December and the sum of effective temperatures in September of the preceding season) were found to have a significant negative effect on the root growth rate of plantain seedlings from both zones. Coefficients *b*_0_ and *b*_1_ of linear regression equations for both zones were unidirectional and similar (see Table 3). Therefore, the magnitudes of influence of these factors on the root growth rate of plantain progeny were probably similar between the different zones. The main difference in the effects of weather conditions on *P. major* seed progeny quality is that in the background groups, 88.9% of the effects were negative, while in the impact groups, 78.5% were positive. It must be noted that the viability of *P. major* seed progeny was affected not only by factors characterizing weather conditions of the current spring-summer season, but also by conditions of the autumn-winter period of the preceding year.

### 2.6. The Influence of Weather Parameters on Radioresistance of Seed Progeny

In the background groups of seeds, there were no significant correlations between weather conditions and the seedling survival rate or root length after the provocative irradiation at the dose of 100 Gy. After 200 Gy irradiation, the seedling survival rate of the background groups negatively correlated with the *HTC* for April of the current year and for September of the preceding year (Table 4). There were no significant effects of weather conditions on the root length of plantain seedlings from background populations.

After the acute irradiation, a negative correlation of root length with weather conditions was demonstrated in impact groups. The strongest root response was recorded under abundant precipitation during seed formation (see Table 4). The sum of effective temperatures from June to August positively correlated with the root length of seedlings from the EURT zone after the 200 Gy irradiation. In other words, high temperatures during the growth and flowering of mother plants as well as seed maturation contributed to their greater resistance.

The provocative irradiation revealed latent variability in seedling survival and root length in seed progeny arising in the different zones. There were no identical weather factors influencing the radioresistance of plantain seedlings if we compared the background and impact groups. The greater number of significant correlations in plantain seed progeny from the EURT zone points to its higher susceptibility to changing weather conditions as compared with that of the background groups.

## 3. Discussion

Dose rates for parental *P. major* plants at the EURT sites ranged from 14.5 to 165.9 μGy h^−1^ and corresponded to low doses for plants [25,26]. The International Commission on Radiological Protection defines the dose rate of 41.7–417 µGy h^−1^ as the range within which potential radiobiological effects can be observed in herbaceous plants [27]. Dose rates for *P. major* plants are within this range at the two most contaminated sites in the head part of the EURT. The U.S. Department of Energy defines the dose rate of 400 µGy h^−1^ as the threshold for a search for radiobiological effects [28]. It is worth mentioning that the historical doses received by previous generations of *P. major* in the first decades after the Kyshtym accident were high; these doses may have contributed to modern biological effects [6,29].

Observable dose loads in the EURT zone did not lead to stable changes in the quality of seed progeny of greater plantain in comparison with that of background groups. Namely, seed weight, the seedling survival rate, and root length were not affected by dose rates at the sites. Similar data were obtained in a study on the seed mass of *Daucus carota* growing in the zone of influence of the Chernobyl accident at the dose rate of 0.08–30.2 μGy h^−1^ [30] and in a paper on *Arabidopsis thaliana* from the same zone (2.8–99.0 μGy h^−1^) [15]. Therefore, our hypothesis was confirmed: there is no dependence of plantain seed progeny quality on the dose rate absorbed by the parental plants.

We detected no significant correlations between seed weight and the seedling survival rate in the two zones; the same was true for seed weight and root length in the background populations. Effects were found only for root length in the EURT zone. Associations between seed size (weight) and the plant survival rate have been described by other researchers [31]. Our previous paper on white campion from impact populations suggests that in 2010, large seeds had high seedling survival and growth rates, while small seeds had low viability [32]. Moreover, 32.5% of seedlings with the *monopteros* mutation were observed in the small-seed fraction, but only 1% in the large seed fraction [32].

As a result of the analysis of the 12 years of observations, we demonstrated significant interannual variability in the seedling survival rate, seed weight, and root length of greater plantain in background and impact populations. Ranges of variability of these parameters often overlapped between the two zones. Similar effects have been detected in *Pinus sylvestris* from the Chernobyl zone (1.24–4.5 µGy h^−1^) [20]. Its seeds are characterized by high interannual variability in germination and a high proportion of abortive seeds. The relation of these traits with dose loads was not detectable in pine trees either [20]. Various trends of the viability of *Bromus inermis* in the EURT contamination gradient (0.15–9.06 μGy h^−1^) have been documented in different years as compared with that of background populations: an increase, decrease, or no difference [33]. Similar effects have been observed in populations of *Taraxacum officinale* (0.15–9.06 µGy h^−1^) [6] and *Leonurus quinquelobatus* (0.22–70 µGy h^−1^) in the same zone [18]. These research articles allow us to conclude that interannual variability in seed progeny quality is determined by the interaction of low radiation doses with weather conditions; in some cases, weather factors play the leading role in seed formation processes.

The dependence of seed progeny quality on weather conditions was revealed here for *P. major*. Significant correlations with weather parameters were registered only for root length in background plantain groups, whereas in impact groups of the plants, the correlations with weather conditions involved all the studied parameters. Thus, another hypothesis of ours was confirmed: susceptibility of seed progeny of *P. major* to weather factors is greater in the EURT zone than in the background groups. A similar conclusion has been made about *Leonurus quinquelobatus* [18] and *Stellaria graminea* [21] from the same zone. By contrast, the pattern is the opposite for *B. inermis* populations [33]. These observations indicate species specificity of plants’ responses to the combined effects of ionizing radiation and of factors unrelated to radiation. Note also that in both zones, the quality of plantain seed progeny correlated with the weather conditions not only of the current growing season but also of the autumn-winter period of the preceding year. A similar phenomenon in motherwort was revealed earlier [18].

In *P. major* in our work, positive correlations of the sum of precipitation in August with the seedling survival rate, seed weight, and root length were found only in the EURT zone. In addition, a positive correlation of the *HTC* for August with seed weight and root length was registered in impact groups. This finding indicates that for the formation of quality seed progeny of plantain in the EURT zone, the most important factor is precipitation during seed ripening; the effect is positive according to our results. Negative correlations between precipitation in March and June and root length were found in the background groups. The leading role of the water regime has also been highlighted in experiments with *Arabidopsis thaliana* [34]. Namely, growth parameters were significantly influenced by water stress but were only weakly sensitive to high temperature. Similarly, drought but not elevated temperature has been found to alter the metabolism of amino acids, organic acids, and osmolytes and nitrogen assimilation in the seeds of two *Hordeum sativum* genotypes [35].

It is known that the lack or excess of any factors limiting plant growth triggers various molecular, biochemical, and physiological mechanisms that contribute to homeostasis. Examples of these mechanisms are the synthesis of microRNA; expression of MYB family genes and transcription factors NAC, WRKY, and DREB; and changes in the activity of enzymes CAT, APX, and GR and in the lipid content [36,37,38,39,40]. Moisture deficiency, just as chronic irradiation at low doses, can increase the amount of reactive oxygen species in plant cells, and thereby stimulate the protective action of antioxidant systems [41,42]. Low-dose pre-sowing γ-irradiation can improve resistance to moisture deficiency, as shown in soybeans [43]. Low doses of radiation can affect water metabolism in plants; the evidence is overexpression of the *TIP1* gene (which is involved in water homeostasis) in *Trifolium repens* L. from the Chernobyl zone (8 µGy h^−1^) [4]. The combined effects of irradiation and of different amounts of precipitation under field conditions may be synergistic or antagonistic toward biochemical and physiological processes affecting the quality of seed progeny. Positive correlations of the amount of precipitation (and of the HTC in July and August) with the high quality of plantain seeds from the EURT zone are probably related to this phenomenon.

In this work, when we evaluated viability parameters of seed progeny, acute pre-sowing γ-irradiation of *P. major* seeds did not reveal a stable preadaptation effect in the impact groups. A greater number of correlations between weather factors and seed progeny radioresistance indices (viability parameters after acute irradiation at 100 and 200 Gy) were uncovered in groups from the EURT zone compared with the background groups. This finding implies the expansion of plants’ limits of the response to weather factors under chronic low-dose irradiation. In *Leonurus quinquelobatus*, the effect of weathering factors on seed radioresistance in EURT groups is also reported to be stronger than that in background plants [18]. In that paper, for the majority of interactions of “weather conditions–the response to provocative irradiation,” the effects were positive in the background groups, whereas in the impact groups the effects were negative [18]. These data are in agreement with a study on spring wheat varieties of different radioresistance [44]. That report shows that the damage from radiation increases during a drought period in the growing season.

The greater number of correlations of weather factors with parameters of the quality of seed progeny (and with the viability of seedlings after provocative irradiation) indicates an increase in the diversity of plant responses under low doses of irradiation. A change in the norm of the reaction of herbaceous plants has been documented for other types of anthropogenic contamination too. For example, in populations of *Crepis tectorum* in habitats polluted with acid-soluble fluorine compounds, an alteration of phenotypic plasticity has been revealed that depends on temperature [45]. In natural populations of plants growing in environments subject to anthropogenic pollution, the assessment of the modifying effect of weather factors on biological effects is becoming especially relevant during climate change.

## 4. Materials and Methods

### 4.1. Materials

Greater plantain (*P. major* L.) is a perennial herbaceous polycarpic plant. It is commonly found in meadows and along roadsides. It is a hemicryptophyte and mesotroph [46,47]. This species reproduces via seeds and has high seed production [48]. *P. major* is sensitive to ionizing irradiation; the median lethal dose (LD_50_) in air-dry seeds is estimated by various authors to be 75 Gy [49] to 100 Gy [50].

### 4.2. The Study Area

On September 29, 1957, a tank with radioactive waste exploded at Mayak Production Association (the so-called Kyshtym accident). Approximately 7.4 × 10^17^ Bq of radioactive material was released into the environment: ~10% of it rose into the atmosphere and formed the EURT while descending from the radioactive cloud to the ground [51]. Short-lived radionuclides constituted the bulk of the isotope mixture; they decayed during the first 4 years after the accident. Over time, the radiation background in the contaminated area decreased as radioactive decay proceeded, and the radiation became chronic, low-intensity [51]. Additional contamination of the EURT area with ^137^Cs (2.22 × 10^13^ Bq) occurred in 1967 as a result of a wind transfer of dried silt and sand from the shores of shallowed Lake Karachay, which was an open storage facility for radioactive waste [52]. At present, the main pollutant of the EURT area is the β-emitter ^90^Sr (half-life [T_1/2_] is 28.6 years). The density of ^90^Sr contamination of soils within the central axis of the EURT reaches 70,000 kBq m^−2^ (the background level in the Ural region is 1.5−3 kBq m^−2^) and diminishes with the distance from the explosion epicenter in accordance with a power law [53]. According to the latest estimates, the bulk of radionuclides (50–60% of ^90^Sr and up to 90% of ^137^Cs from the total amount in the soil profile) are concentrated in the 0–20 cm root layer of the soil [53].

The EURT is located in the forest-steppe zone, and there are two large lakes (Berdenish and Uruskul) in the area: there is also an extensive wetland in the northern part of the EURT. Secondary birch (92%) and mixed forests dominate in the area, and dry and flood meadows are present too. More than 200 species of herbaceous plants grow in the area, and major species are cereals [54]. In the EURT zone, *P. major* mainly occurs on roadsides and between ruts of rarely used dirt roads. The soil cover is dominated by various subtypes of gray forest soils and chernozem soils of varying thicknesses and degrees of leaching; various brown forest and peaty soils occur as well [54].

**Figure 5:**
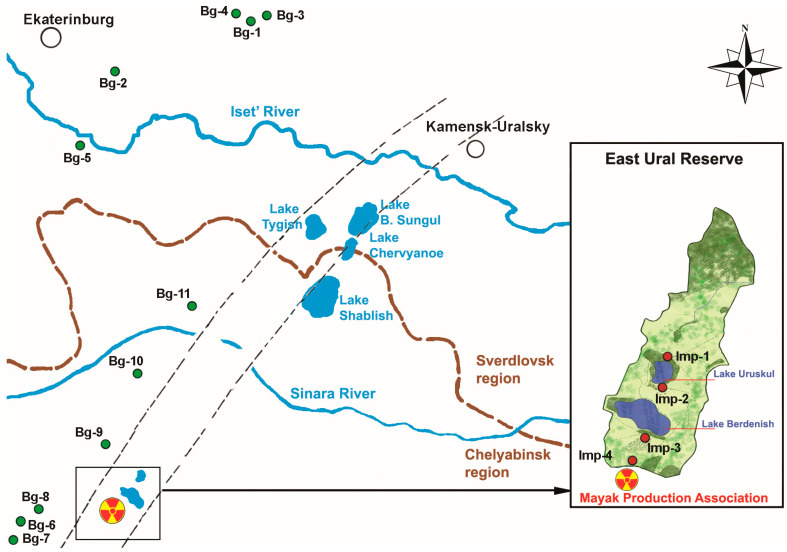
A map of the study area (adapted from [55]).

The background sites (control groups) that were similar in geobotanical characteristics and soil conditions were selected outside the EURT and industrial impact zones (Figure 5). Three to ten background and impact sites were investigated in different years.

### 4.3. Collection of Seeds

According to the methodology of impact region research [56], the study sites were chosen at different distances from the epicenter of the Kyshtym accident. Seeds were collected in 2005–2021 (except for 2006−2009 and 2012) on reference plots from 40−50 *P. major* plants along transects 0.5 to 1 km long that were associated with roadsides of rarely used country roads or cuttings. Seeds were always collected at full maturity in late August from plants in the middle generative stage of development.

### 4.4. Calculation of Dose Rates on Parental Plants of P. major

Absorbed dose rates were calculated in the ERICA Tool software (Tier 2) [57]. Empirical data on concentrations of the main dose-forming radionuclides (^90^Sr and ^137^Cs) in soil and in vegetative mass of plants were used for the calculations. Methods for estimating ^90^Sr and ^137^Cs concentrations, model assumptions, and calculations are described in detail in ref. [25]. The results of calculations (Table 5) were normalized to certain years under study (taking into account ^90^Sr and ^137^Cs half-lives). Compared with 2005, current dose loads of parental plantain plants decreased by 33% in the EURT zone.

### 4.5. Pre-Sowing Treatment and Estimation of 1000-Seed Weight

The seeds were dried in the laboratory, cleansed of impurities, and stored in paper bags at 4 °C until the experiments. One hundred air-dried *P. major* seeds were randomly selected from each coenopopulation in five replicates and then weighed on an analytical balance (Kern 770, Balingen, Germany). The weight was calculated per 1000 seeds.

### 4.6. Laboratory Germination of Seeds and Selection of Parameters for the Evaluation of Various Effects

Seeds were germinated the next year after collection (in March–July) using the roll culture method in glass vessels on distilled water for 21 days (12 h photoperiod, at 24 °C). From each group of seeds, 25−50 seeds were randomly chosen and sown in 3−4 replicates. A total of 26,350 seeds (800 to 4800 seeds in different years) were used in the experiments.

The viability of seed progeny was evaluated by means of the following parameters: (1) the seed germination rate (% of the sown seeds); (2) the survival rate of seedlings (% of the sown seeds); (3) the survival rate of seedlings at the stage of leaf formation (the proportion of live seedlings having a true leaf, % of the sown seeds); (4) the leaf formation rate (the proportion of seedlings with a true leaf, % of surviving seedlings); (5) the proportion of seedlings with two or more leaves (% of the surviving seedlings); (6) primary root length (mm); and (7) the proportion of seedlings with lateral roots (% of the surviving seedlings).

Correlation analysis uncovered a strong association between the following parameters (presented as pairs) of seed viability: the seed germination rate and the survival rate (*R* = 0.99; *p* < 0.001); root length and the leaf formation rate (*R* = 0.87−0.88; *p* < 0.001); root length and the proportion of seedlings with lateral roots (*R* = 0.87−0.90; *p* < 0.001); the leaf formation rate and the proportion of seedlings with lateral roots (*R* = 0.87−0.91; *p* < 0.001); and the survival rate of seedlings at the stage of leaf formation (*R* = 0.88−0.90; *p* < 0.001). Significant correlations of the survival rate of seedlings at the stage of leaf formation with root length (*R* = 0.46; *p* = 0.004) and with the leaf formation rate (*R* = 0.45; *p* = 0.004) were observed in the EURT combined group. In the background area, the correlations between these parameters were not significant. Via this analysis, we identified the two most informative parameters: the survival rate of seedlings at the stage of leaf formation (codenamed as “survival rate”) and root length. These parameters have prognostic value because they characterize the functioning of apical meristems of seedlings and are most convenient for estimating seed progeny viability.

### 4.7. Assessment of Seed Progeny Radioresistance

The radioresistance of *P. major* was assessed in laboratory experiments. Irradiation of air-dried seeds was carried out on a γ-installation with a ^137^Cs source at a power dose rate of 38–44 Gy s^−1^. The doses of 100 or 200 Gy were employed. These doses were chosen based on an analysis of the detailed dose-effect curve described earlier [50]. Radioresistance was evaluated by means of the seedling survival rate and root length, with a comparison to these values in an identical unirradiated control.

### 4.8. Weather Data Collection and Calculation of Weather Indices

A database containing data on the 12 years of observation (2005, 2010, 2011, and 2013–2021) was created for the analysis of the weather conditions’ influence on *P. major* seed quality and seed-progeny radioresistance. Weather data from two local meteorological stations (#28541 and #28440) located near the study sites were utilized to calculate weather parameters. The set of parameters tested earlier on other plant species was used for the assessment of weather conditions in different years [18]. We chose the factors most physiologically important for plants: sums of effective temperatures (average daily temperatures above 10 °C), precipitation amounts, precipitation amounts at effective temperatures, and the *HTC* calculated via the formula:

(1) $$HTC=\frac{P\times 10}{\sum T_{n}}$$

where *P* is the sum of precipitation at effective temperatures for a given month, and Σ*T_n_* is the sum of effective temperatures for the same period [58]. The aforementioned indices were calculated for each month; data from September to December were used for the previous year, because seeds were collected at the end of August. For the examining of the biological parameters’ correlations with weather conditions, groups of seeds were pooled within the background zone and within the impact zones. The main weather parameters (temperature, precipitation, and their ratio in the summer period) characterizing different years in the study region are given in Table A1.

### 4.9. Statistical Hypothesis Testing

For this purpose, we used nonparametric procedures: the Mann–Whitney (*U*) test and the Kruskal–Wallis (*H*) test; for multiple comparisons, Dunn’s test was performed. The significance level (*p*-value) was set to ≤0.05. We also carried out correlation and regression analyses; correlation and linear regression coefficients were considered significant at *p* ≤ 0.01. Calculations were performed in the STATISTICA 10.0 software (Tulsa, OK, USA) [59]. Microsoft Excel and the R programming environment were used for data visualization [60].

## 5. Conclusions

The 12-year monitoring research on the quality of *P. major* seed progeny from radioactively contaminated and background habitats revealed that interannual variability in seed mass, seedling viability, and root length was high. Chronic exposure at dose rates 14.5–165.9 μGy h^−1^ did not worsen the quality of seed progeny of *P. major*, but increased its susceptibility to weather conditions and expanded intraspecific variability in the studied traits. Among the analyzed weather conditions determining the quality of plantain seed progeny, the amount of precipitation was found to play the biggest role in this regard. In background groups of seeds, the majority of biological responses negatively correlated with weather factors, whereas in impact groups, the effects were mostly positive. Radioresistance of greater plantain seeds from the EURT zone depended on weather conditions during the period of seed formation, and there were few significant correlations in the background groups of seeds. Our findings indicate that during research on radiobiological effects in natural populations of living organisms, it is necessary to take into account that the observed effects vary among different years. Therefore, long-term monitoring studies are needed to identify the causes of the variability in radiobiological effects in natural populations. This approach should be interesting and useful for future studies on markers of pro- and antioxidant status and on epigenetic stress memory in plants growing in radioactively contaminated areas.

## Figures and Tables

**Table 1:** Effects of pre-sowing irradiation of seeds by us at a dose of 100 Gy on the main viability parameters of *P. major* seed progeny from background and impact groups in different years.

Year	Survival Rate		Root Length	
	Background	EURT	Background	EURT
2005	Bg-2≈	**Imp-1↗**; Imp-2≈; Imp-2≈	Bg-2≈	**Imp-1↘**; **Imp-2↘**; Imp-3≈
2010	Bg-2≈; Bg-3≈	Imp-2-1≈; Imp-2-2≈; Imp-3≈	Bg-2≈; Bg-3≈	Imp-2-1≈; Imp-2-2≈; Imp-3≈
2011 *	**Bg-2↘**; Bg-3≈	**Imp-1↘**; **Imp-2↘**	**Bg-2↘**; Bg-3≈	**Imp-1↘**; **Imp-2↘**
2013	Bg-2≈; Bg-4≈	Imp-4≈	**Bg-2↘**; **Bg-4↘**	**Imp-4** **↘**
2014	Bg-2≈; Bg-3≈; Bg-4≈ Bg-5-1≈; Bg-5-2≈	**Imp-2↘**; **Imp-3-1↘**; Imp-3-2≈; Imp-3-3≈; Imp-4≈	Bg-2≈; **Bg-3↘**; **Bg-4↘**; **Bg-5-1↘**; **Bg-5-2↘**	**Imp-2↘**; **Imp-3-1↘**; **Imp-3-2↘**; **Imp-3-3↘**; **Imp-4↘**
2015	**Bg-3↘**; Bg-5≈; **Bg-6↘**; **Bg-7↘**	**Imp-2↘**; **Imp-3↘**; Imp-4≈	**Bg-3↘**; **Bg-5↘**; Bg-6≈; **Bg-7↘**	**Imp-2↘**; **Imp-3↘**; **Imp-4↘**
2016	**Bg-2↘**; **Bg-3↘**; **Bg-5↘**; Bg-6≈; Bg-7≈	Imp-2≈; Imp-3≈; Imp-4≈	**Bg-2↘**; **Bg-3↘**; **Bg-5↘**; Bg-6≈; **Bg-7↘**	Imp-2≈; **Imp-3↘**; **Imp-4↘**
2017	**Bg-3↘**; **Bg-5↘**; **Bg-7↘**; **Bg-8↘**	**Imp-2↘**; **Imp-3↘**; **Imp-4↘**	**Bg-3↘**; **Bg-5↘**; **Bg-7↘**; **Bg-8↘**	**Imp-2↘**; **Imp-3↘**; **Imp-4↘**
2018*	**Bg-7↘**; **Bg-8↘**	Imp-2≈; **Imp-3-1↘**; **Imp-3-2↘**; **Imp-4↘**;	**Bg-7↘**; **Bg-8↘**	**Imp-2↘**; **Imp-3-1↘**; **Imp-3-2↘**; **Imp-4↘**
2020	**Bg-3↘**; **Bg-9↘**; **Bg-10↘**; **Bg-11↘**	**Imp-2↘**; **Imp-3-1↘**; **Imp-3-2↘**; **Imp-4↘**	**Bg-3↘**; **Bg-9↘**; **Bg-10↘**; **Bg-11↘**	**Imp-2↘**; **Imp-3-1↘**; **Imp-3-2↘**; **Imp-4↘**
2021	**Bg-9↘**; **Bg-10↘**; **Bg-11↘**	**Imp-2↘**; **Imp-3↘**; **Imp-4↘**	**Bg-9↘**; **Bg-10↘**; **Bg-11↘**	**Imp-2↘**; **Imp-3↘**; **Imp-4↘**

Note: Significant differences from the unirradiated control are indicated by boldfacing and arrows (↘: inhibition, ↗: stimulation, ≈: no difference according to the *U* test, *p* < 0.001 to 0.048, * at n = 3, the *H*-test was applied instead, *p* < 0.001 to 0.035).

**Table 2:** Correlation coefficients (*R*) and analysis of linear regression equations *y* = *b*_0_ + *b*_1_*x* for 1000-seed weight of *P. major* from the EURT area.

**Weather Factor**	***R***	***p*-Value**	***b*_0_ ± SE**	***b*_1_ ± SE**	**n**
Σ*P*_8_	0.45	0.0050	0.2233 ± 0.0072	0.0004 ± 0.0001	38
Σ*P*_10_	0.42	0.0088	0.2239 ± 0.0075	0.0003 ± 0.0001	38
Σ*P*-*Tef*_10_	0.81	0.0044	0.2214 ± 0.0071	0.0038 ± 0.0010	11
*HTC*_8_	0.48	0.0029	0.2242 ± 0.0066	0.0171 ± 0.0053	38

Note. Σ*P*: the sum of precipitation, *HTC*: Selyaninov hydrothermal coefficient, Σ*P*-*Tef*: the sum of precipitation at effective temperatures, n: the number of observations. The subscripts in the names of weather factors denote months. Weather factors from September to December characterize the previous season. Coefficients *b*_0_ and *b*_1_ are significant at *p* < 0.01.

**Table 3:** Correlation coefficients (*R*) and analysis of linear regression equations *y* = *b*_0_ + *b*_1_*x* for viability parameters of *P. major* seed progeny from the impact and background zones.

Viability Parameter	Weather Factor	*R*	*p*-Value	*b*_0_ ± SE	*b*_1_ ± SE	n
Background populations						
Root length, mm	Σ*P*_3_	−0.44	0.0056	61.81 ± 5.47	−0.49 ± 0.17	38
	Σ*P*_6_	−0.52	0.0009	66.78 ± 5.85	−0.25 ± 0.07	38
	Σ*P*_11_	−0.55	0.0003	64.16 ± 4.78	−0.46 ± 0.11	38
	Σ*P*_12_	−0.61	<0.0001	67.93 ± 4.98	−0.63 ± 0.14	38
	Σ*Tef*_9_	−0.59	0.0001	75.28 ± 6.77	−0.14 ± 0.03	38
	Σ*P*-*Tef*_6_	−0.53	0.0006	64.15 ± 5.03	−0.23 ± 0.06	38
	Σ*P*-*Tef*_9_	−0.45	0.0047	60.07 ± 4.85	−0.93 ± 0.31	38
	*HTC*_4_	0.81	0.0015	26.43 ± 5.66	28.52 ± 6.57	12
	*HTC*_6_	−0.47	0.0029	63.20 ± 5.51	−10.13 ± 3.18	38
	Σ*P*_3_	−0.44	0.0056	61.81 ± 5.47	−0.49 ± 0.17	38
	Σ*P*_6_	−0.52	0.0009	66.78 ± 5.85	−0.25 ± 0.07	38
Impact populations						
Survival rate, %	Σ*P*_8_	0.42	0.0083	16.59 ± 6.19	0.30 ± 0.11	38
	Σ*Tef*_4_	−0.50	0.0025	43.24 ± 4.65	−0.35 ± 0.11	34
	Σ*P*-*Tef*_8_	0.47	0.0027	15.49 ± 5.81	0.34 ± 0.11	38
Root length, mm	Σ*P*_8_	0.51	0.0011	27.31 ± 5.02	0.31 ± 0.09	38
	Σ*P*_12_	−0.45	0.0042	61.89 ± 6.67	−0.59 ± 0.19	38
	Σ*Tef*_9_	−0.51	0.0011	75.03 ± 9.34	−0.20 ± 0.06	38
	Σ*P*-*Tef*_7_	0.44	0.0050	23.87 ± 6.88	0.23 ± 0.08	38
	Σ*P*-*Tef*_8_	0.52	0.0009	27.81 ± 4.83	0.32 ± 0.09	38
	*HTC*_7_	0.43	0.0069	23.32 ± 7.32	12.07 ± 4.21	38
	*HTC*_8_	0.56	0.0002	27.54 ± 4.50	14.71 ± 3.65	38

Note. Σ*Tef*: the sum of effective temperatures, Σ*P*: the sum of precipitation, *HTC*: the Selyaninov hydrothermal coefficient, Σ*P*-*Tef*: the sum of precipitation at effective temperatures, n: the number of observations. The subscripts in the names of weather factors denote months. Weather factors from September to December characterize the preceding season. Coefficients *b*_0_ and *b*_1_ are significant at *p* < 0.01.

**Table 4:** Correlation coefficients (*R*) and analysis of linear regression equations *y* = *b*_0_ + *b*_1_*x* for viability parameters of *P. major* seed progeny from impact and background zones after pre-sowing irradiation of seeds at the dose of 100 or 200 Gy.

Viability Parameter	Weather Factor	*R*	*p*-Value	*b*_0_ ± SE	*b*_1_ ± SE	n
Impact populations, 100 Gy						
Survival rate, %	Σ*P*-*Tef*_4_	−0.79	0.0014	40.44 ± 7.28	−9.91 ± 2.35	13
Root length, mm	Σ*P*_4_	−0.62	0.0001	11.96 ± 1.75	−0.20 ± 0.04	33
	Σ*P*_7_	−0.52	0.0019	13.60 ± 2.70	−0.09 ± 0.03	33
	ΣP-*Tef*_6–8_	−0.48	0.0044	14.45 ± 3.22	−0.05 ± 0.01	33
	*HTC*_6–8_	−0.44	0.0097	12.62 ± 2.93	−5.61 ± 2.03	33
Background populations, 200 Gy						
Survival rate, %	*HTC*_4_	−0.84	0.0093	22.84 ± 5.60	−24.52 ± 6.50	8
	*HTC*_9_	−0.49	0.0092	18.10 ± 5.21	−18.80 ± 6.66	27
Impact populations, 200 Gy						
Root length, mm	Σ*P*_6_	−0.68	0.0001	11.72 ± 1.91	−0.12 ± 0.03	23
	Σ*P*_7_	−0.66	0.0003	12.91 ± 2.28	−0.10 ± 0.02	23
	Σ*P*_8_	−0.50	0.0095	7.69 ± 1.63	−0.08 ± 0.03	23
	Σ*P*-*Tef*_6_	−0.57	0.0022	9.45 ± 1.84	−0.10 ± 0.03	23
	Σ*P*-*Tef*_8_	−0.53	0.0057	7.73 ± 1.55	−0.09 ± 0.03	23
	*HTC*_6_	−0.67	0.0002	11.09 ± 1.81	−5.29 ± 1.20	23
	*HTC*_7_	−0.51	0.0082	9.75 ± 2025	−3.73 ± 1.30	23
	*HTC*_8_	−0.54	0.0042	7.62 ± 1.47	−3.77 ± 1.19	23
	Σ*Tef*_6–8_	0.81	<0.0001	−37.03 ± 6.12	0.03 ± 0.004	23
	Σ*P*-*Tef*_6–8_	−0.74	0.0042	15.98 ±2.36	−0.06 ± 0.01	23
	*HTC*_6–8_	−0.76	<0.0001	14.73 ± 2.05	−8.04 ± 1.42	23

Note. Σ*Tef*: the sum of effective temperatures, Σ*P*: the sum of precipitation, *HTC*: the Selyaninov hydrothermal coefficient, Σ*P*-*Tef*: the sum of precipitation at effective temperatures, n: the number of observations. The subscripts in the names of weather factors denote months. Weather factors from September to December characterize the preceding season. Coefficients *b*_0_ and *b*_1_ are significant at *p* < 0.01.

**Table 5:** Ranges of absorbed dose rates (ADR) of parental *P. major* plants in the EURT zone during the years under study.

Site	Site Name	GPS Coordinates	ADR, μGy h^−1^ *	Multiplicity Factor of Background Level Excess
Background values	Bg	-	0.091−0.129	1
Northern shore of Lake Uruskul	Imp-1	55°49′ N, 60°55′ E	14.51−16.72 **	145−167
Southwest shore of Lake Uruskul	Imp-2	55°49′ N, 60°55′ E	16.94−24.75	169−248
South shore of Lake Berdenish	Imp-3	55°46′ N, 60°52′ E	64.01−93.63	640−936
Roadway at southern boundary of EURT	Imp-4	55°44′ N, 60°50′ E	137.16−165.90	1371−1659

* Absorbed dose rates were calculated taking into account natural background radiation, which is ~0.088 μGy h^−1^ in the Ural region. Cited from ref. [25] with updates and additions (** own data).

## Data Availability

Raw data are available upon request.

## Appendix A

**Table A1:** The main weather parameters characterizing the temperature, precipitation, and their ratio in the summer period in the study region. The data were borrowed from two weather stations.

Year	Σ*Tef*_6–8_	Σ*P*-*Tef*_6–8_	*HTC*_6–8_
#28541 (Verkhny Ufaley)			
2005	1370.5	182.7	1.33
2010	1648.5	55.3	0.34
2011	1321.1	254.8	1.93
2013	1502.7	151.3	1.01
2014	1292.1	221.0	1.71
2015	1321.4	249.4	1.89
2016	1645.8	140.6	0.85
2017	1395.8	206.4	1.48
2018	1317.1	156.5	1.19
2019	1338.6	215.5	1.61
2020	1435.4	222.5	1.55
2021	1573.5	203.2	1.29
Mean	1423.2	190.4	1.37
#28440 (Ekaterinburg)			
2005	1542.3	357.4	2.32
2010	1765.1	262.3	1.49
2011	1530.3	347.6	2.27
2013	1715.1	147.8	0.86
2014	1400.2	255.2	1.82
2015	1409.4	247.2	1.75
2016	1866.7	100.0	0.54
2017	1554.1	256.8	1.65
2018	n.o. *	n.o.	n.o.
2019	1531.5	248.0	1.62
2020	1632.3	146.9	0.90
2021	n.o.	n.o.	n.o.
Mean	1594.7	236.9	1.52

Note: Σ*Tef*_6–8_ is the sum of effective temperatures for the summer months, Σ*P*-*Tef*_6–8_ is the sum of precipitation at effective temperatures for the summer months, *HTC*_6–8_ is the Selyaninov hydrothermal coefficient for the summer months. * In 2018 and 2021, no greater plantain seeds were collected at sites near weather station #28440.

**Table A2:** Results of multiple comparisons of root length using Dunn’s post hoc test among seeds harvested 2005 in the background and impact areas. Below the diagonal are *p*-values, with asterisks (*) indicating significant differences (*p* < 0.05); above the diagonal are *z*-values.

2005	Bg-2	Imp-1	Imp-2	Imp-3
Bg-2		5.577	4.915	4.575
Imp-1	<0.001 *		0.551	9.915
Imp-2	<0.001 *	1.000		9.193
Imp-3	<0.001 *	<0.001 *	<0.001 *	

**Table A3:** Results of multiple comparisons of root length using Dunn’s post hoc test among seeds harvested in 2011 in the background and impact areas. Below the diagonal are *p*-values, with asterisks (*) indicating significant differences (*p* < 0.05); above the diagonal are *z*-values.

2011	Bg-2	Bg-3	Imp-1	Imp-2
Bg-2		4.264	6.179	1.238
Bg-3	<0.001 *		2.378	7.121
Imp-1	<0.001 *	0.105		9.574
Imp-2	1.000	<0.001 *	<0.001 *	

**Table A4:** Results of multiple comparisons of root length using Dunn’s post hoc test among seeds harvested in 2013 in the background and impact areas. Below the diagonal are *p*-values, with asterisks (*) indicating significant differences (*p* < 0.05); above the diagonal are *z*-values.

2013	Bg-2	Bg-4	Imp-4
Bg-2		1.306	2.613
Bg-4	0.575		4.012
Imp-4	0.027 *	<0.001 *	

**Table A5:** Results of multiple comparisons of root length using Dunn’s post hoc test among seeds harvested in 2014 in the background and impact areas. Below the diagonal are *p*-values, with asterisks (*) indicating significant differences (*p* < 0.05); above the diagonal are *z*-values.

2014	Bg-2	Bg-3	Bg-4	Bg-5-1	Bg-5-2	Imp-2	Imp-3-1	Imp-3-2	Imp-3-3	Imp-4
Bg-2		6.630	2.177	2.490	0.145	2.911	5.399	6.564	1.369	1.523
Bg-3	<0.001 *		5.185	5.142	7.305	4.268	1.481	1.361	8.061	8.607
Bg-4	1.000	<0.001 *		0.254	2.450	0.914	3.785	4.862	3.795	4.235
Bg-5-1	0.575	<0.001 *	1.000		2.871	0.715	3.704	4.847	4.202	4.734
Bg-5-2	1.000	<0.001 *	0.642	0.184		3.274	6.018	7.716	1.768	2.034
Imp-2	0.162	0.001 *	1.000	1.000	0.048 *		2.850	3.679	4.477	4.934
Imp-3-1	<0.001 *	1.000	0.007 *	0.010 *	<0.001 *	0.197		0.316	6.894	7.437
Imp-3-2	<0.001 *	1.000	<0.001 *	<0.001 *	<0.001 *	0.011 *	1.000		8.411	9.336
Imp-3-3	1.000	<0.001 *	0.007 *	0.001 *	1.000	<0.001 *	<0.001 *	<0.001 *		0.040
Imp-4	1.000	<0.001 *	0.001 *	<0.001 *	1.000	<0.001 *	<0.001 *	<0.001 *	1.000	

**Table A6:** Results of multiple comparisons of root length using Dunn’s post hoc test among seeds harvested in 2015 in the background and impact areas. Below the diagonal are *p*-values, with asterisks (*) indicating significant differences (*p* < 0.05); above the diagonal are *z*-values.

2015	Bg-3	Bg-5	Bg-6	Bg-7	Imp-2	Imp-3	Imp-4
Bg-3		1.884	0.919	0.779	0.851	2.469	2.943
Bg-5	1.000		2.246	2.405	1.096	0.843	0.944
Bg-6	1.000	0.518		0.287	1.505	2.732	3.018
Bg-7	1.000	0.340	1.000		1.517	2.898	3.359
Imp-2	1.000	1.000	1.000	1.000		1.808	2.130
Imp-3	0.284	1.000	0.132	0.079	1.000		0.042
Imp-4	0.068	1.000	0.053	0.016 *	0.697	1.000	

**Table A7:** Results of multiple comparisons of root length using Dunn’s post hoc test among seeds harvested in 2016 in the background and impact areas. Below the diagonal are *p*-values, with asterisks (*) indicating significant differences (*p* < 0.05); above the diagonal are *z*-values.

2016	Bg-2	Bg-3	Bg-5	Bg-6	Bg-7	Imp-2	Imp-3	Imp-4
Bg-2		1.826	2.569	0.442	0.801	3.074	2.139	1.871
Bg-3	1.000		0.528	0.986	2.458	4.257	3.740	0.770
Bg-5	0.286	1.000		1.463	3.187	4.841	4.610	0.478
Bg-6	1.000	1.000	1.000		1.037	2.957	2.046	1.390
Bg-7	1.000	0.391	0.040 *	1.000		2.391	1.222	2.286
Imp-2	0.059	0.001 *	<0.001 *	0.087	0.470		1.515	3.727
Imp-3	0.908	0.005 *	<0.001 *	1.000	1.000	1.000		3.041
Imp-4	1.000	1.000	1.000	1.000	0.622	0.005 *	0.066	

**Table A8:** Results of multiple comparisons of root length using Dunn’s post hoc test among seeds harvested in 2017 in the background and impact areas. Below the diagonal are *p*-values, with asterisks (*) indicating significant differences (*p* < 0.05); above the diagonal are *z*-values.

2017	Bg-3	Bg-5	Bg-7	Bg-8	Imp-2	Imp-3	Imp-4
Bg-3		4.193	2.828	1.724	2.948	2.467	3.013
Bg-5	0.001 *		1.327	2.870	1.443	2.127	1.663
Bg-7	0.098	1.000		1.388	0.040	0.653	0.168
Bg-8	1.000	0.086	1.000		1.439	0.831	1.413
Imp-2	0.067	1.000	1.000	1.000		0.653	0.134
Imp-3	0.286	0.702	1.000	1.000	1.000		0.564
Imp-4	0.054	1.000	1.000	1.000	1.000	1.000	

**Table A9:** Results of multiple comparisons of root length using Dunn’s post hoc test among seeds harvested in 2018 in the background and impact areas. Below the diagonal are *p*-values, with asterisks (*) indicating significant differences (*p* < 0.05); above the diagonal are *z*-values.

2018	Bg-7	Bg-8	Imp-2	Imp-3-1	Imp-3-2	Imp-4
Bg-7		0.512	1.119	0.290	0.714	3.317
Bg-8	1.000		0.624	0.709	0.169	2.571
Imp-2	1.000	1.000		1.227	0.490	1.652
Imp-3-1	1.000	1.000	1.000		0.880	3.016
Imp-3-2	1.000	1.000	1.000	1.000		2.502
Imp-4	0.014 *	0.152	1.000	0.038 *	0.185	

**Table A10:** Results of multiple comparisons of root length using Dunn’s post hoc test among seeds harvested in 2020 in the background and impact areas. Below the diagonal are *p*-values, with asterisks (*) indicating significant differences (*p* < 0.05); above the diagonal are *z*-values.

2020	Bg-3	Bg-9	Bg-10	Bg-11	Imp-2	Imp-3-1	Imp-3-2	Imp-4
Bg-3		6.731	9.651	3.157	8.096	6.141	10.011	7.291
Bg-9	<0.001 *		2.951	3.142	1.310	0.441	3.922	0.491
Bg-10	<0.001 *	0.089		5.857	1.678	3.311	1.288	2.498
Bg-11	0.045 *	0.047 *	<0.001 *		4.378	2.667	6.537	3.627
Imp-2	<0.001 *	1.000	1.000	<0.001 *		1.720	2.792	0.829
Imp-3-1	<0.001 *	1.000	0.026 *	0.215	1.000		4.233	0.925
Imp-3-2	<0.001 *	0.002*	1.000	<0.001 *	0.147	0.001 *		3.525
Imp-4	<0.001 *	1.000	0.350	0.008 *	1.000	1.000	0.012 *	

**Table A11:** Results of multiple comparisons of root length using Dunn’s post hoc test among seeds harvested in 2021 in the background and impact areas. Below the diagonal are *p*-values, with asterisks (*) indicating significant differences (*p* < 0.05); above the diagonal are *z*-values.

2021	Bg-10	Bg-11	Bg-9	Imp-2	Imp-3	Imp-4
Bg-10		5.936	2.884	5.257	7.244	7.223
Bg-11	<0.001 *		2.967	0.832	1.042	0.793
Bg-9	0.059	0.045 *		2.207	4.108	3.953
Imp-2	<0.001 *	1.000	0.410		1.924	1.696
Imp-3	<0.001 *	1.000	0.001 *	0.816		0.285
Imp-4	<0.001 *	1.000	0.001 *	1.000	1.000	
